# D614G and SARS-CoV-2 replication fitness

**DOI:** 10.1038/s41392-021-00498-3

**Published:** 2021-03-01

**Authors:** Kathleen D. Engelman, Alan N. Engelman

**Affiliations:** 1 MassBiologics, University of Massachusetts Medical School, Boston, MA USA; 2 Department of Cancer Immunology and Virology, Dana-Farber Cancer Institute, Boston, MA USA; 3 Department of Medicine, Harvard Medical School, Boston, MA USA

**Keywords:** Vaccines, Infectious diseases

Molecular determinants of SARS-CoV-2 replication fitness and sensitivity to neutralization via the humoral immune response critically inform efforts to combat the COVID-19 pandemic. Work published recently by Plante et al. in *Nature*^1^ highlights a key role for the D614G variant of the viral spike glycoprotein (S protein) in these processes.

Because major vaccine efforts have employed a D614 reference strain that arose comparatively early,^2^ speculation has focused on potential impacts of genetic variation on viral replication fitness and vaccine development. Although thousands of viral variants have been identified, few have become fixed in circulating populations, with the vast majority bearing inconsequential polymorphisms. However, evidence is mounting that a variant carrying several non-synonymous mutations including the S protein D614G change increases virus transmission.

Most experts agree that effective SARS-CoV-2 vaccination will require generation of neutralizing antibodies that prevent infection by binding to the S protein to sterically block its access of the cellular angiotensin-converting enzyme 2 (ACE2) receptor. For this reason, research has intensively focused on the effect of the D614G change on SARS-CoV-2 fitness and neutralization sensitivity. The Plante et al. study offers compelling data that the G614 virus is more infectious in cells of the upper airway, but still vulnerable to cross-neutralization by convalescent serum from subjects infected with the D614 strain, suggesting that antibodies raised against the ancestral S protein offer protection against its G614 descendant.^1^ This study corroborates similar findings from other groups that consistently demonstrate equal or greater neutralization potency against the G614 variant by polyclonal antiserum or therapeutic monoclonal antibodies, thus easing concerns of a major overhaul of current vaccine development plans.^2–5^

**Fig. 1:**
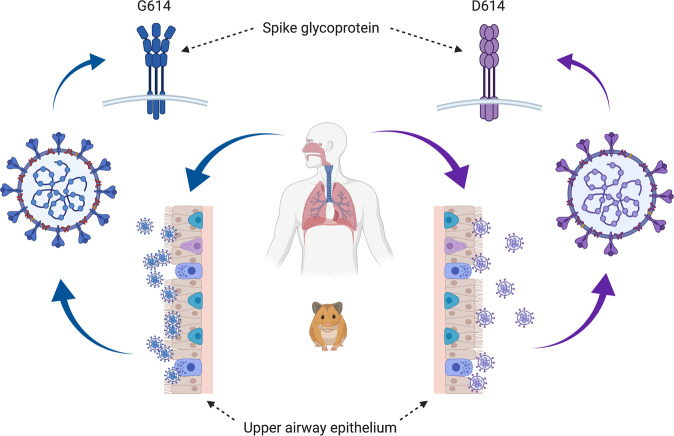
D614G and SARS-CoV-2 transmissibility. The G614 virus variant (left) replicates more efficiently than the ancestral D614 virus in tissues of the upper respiratory epithelium. The higher density of S protein trimers on the viral surface as well as the frequency of spikes in the open receptor-binding configuration may contribute to increased transmission of the D614G mutant virus

Plante et al. present further evidence about the fitness advantage conferred by the D614G change (Fig. 1). While others have presented data suggestive of greater replication fitness of the G614 variant, particularly in cell lines expressing high levels of ACE2,^2–5^ Plante et al. offered several distinctive insights. First, the authors passaged replication-competent virus in a primary airway cell culture system that purportedly maintains the ciliated airway infrastructure, permitting virus spread in a controlled setting that is arguably relevant to physiological conditions. When airway cells were infected with an inoculum containing both viruses, G614 outcompeted its ancestor, even when outnumbered by 9:1 at the starting line, clearly demonstrating a replication advantage.

Second, the authors employed an animal model to investigate if the D614G change similarly impacted replication fitness across different regions of the airway. Viral load was quantified in tissues from upper and lower airway following challenge of Syrian hamsters with D614 or G614 virus. These data failed to indicate a significant change in genome copy number – a common viral load benchmark. However, the authors further quantified virus load using a more rigorous plaque forming assay, which, importantly, measured infectious virus. When comparing plaque forming units to genome copy number, the results were more striking in tissues of the upper airway – nasal and tracheal epithelium – though there was no difference in lung tissues. This implies that the D614G change could confer greater transmissibility, because new infections are more likely to arise from virus shed from the upper airway (Fig. 1). Indeed, this data complements another study that looked at transmission efficiency in discordant hamster pairs. Virus was detected earlier in naïve animals co-housed with cage mates infected with the G614 variant than its D614 ancestor, suggesting that the G614 virus is more efficiently transmitted between individuals.^3^

Several groups have investigated the mechanistic basis of the observed increase in replication fitness. Virus entry was enhanced in immortalized cells expressing high levels of the ACE2 receptor, with more modest differences in lung or intestinal cell lines that harbor lower levels of the cell surface receptor, suggesting that receptor-spike binding affinity might play a role. However, there are conflicting reports as to whether the D614G change increases the binding affinity of S protein for ACE2, and the reported affinity gain was subtle.^4,5^ Plante et al. assessed spike stability by imposing a heat challenge on infectious virus. Infectivity decayed at a slower rate for the G614 variant compared to the D614 ancestor, suggesting that the G614 S protein is more stable at febrile temperatures.

The full-length spike trimeric complex is cleaved by a furin protease into S1 receptor-binding and S1/S2 fusion subunits at the cell surface during egress, priming the virus for entry into the next target cell. The S1 subunit contains the receptor binding domain (RBD), which is capable of adopting an “open” or “closed” configuration. While residue 614 is geographically separated from the RBD, structure-based analysis by single-particle cryogenic-electron microscopy revealed that the G614 variant more frequently adopted the open conformation than did the D614 trimer (Fig. 1).^4^ Leveraging the malleability of retroviruses for effective pseudotyping by heterologous envelope glycoproteins, a separate study determined that the D614G change increased the efficiency of S protein processing and virion spike density, while decreasing the extent of S1 shedding from the pseudovirus surface.^5^ Together, these findings suggest that the G614 variant may have adapted for more efficient transmission by loading virions with a greater number of spikes poised to bind the ACE2-lined upper airway (Fig. 1). The open configuration of the G614 trimer as well as its apparent increase in neutralization sensitivity indicates a potential fitness trade-off for the D614G change that tipped SARS-CoV-2 towards faster transmission along with the risk of exposing vulnerable immune targets.

Variant of concern 202012/01 identified recently in the UK harbors eight additional S protein changes beyond D614G. Epidemiologic modeling of 202012/01 has indicated an ~56% increase in transmissibility relative to pre-existing variants,^6^ sparking concern among policymakers and public health officials. Multipronged approaches akin to those discussed above for D614G should shed light on mechanistic bases of 202012/01 fitness, transmissibility, and neutralization sensitivity.
